# Active Circulation of Madariaga Virus, a Member of the Eastern Equine Encephalitis Virus Complex, in Northeast Brazil

**DOI:** 10.3390/pathogens10080983

**Published:** 2021-08-03

**Authors:** Laura H. V. G. Gil, Tereza Magalhaes, Beatriz S. A. S. Santos, Livia V. Oliveira, Edmilson F. Oliveira-Filho, João L. R. Cunha, Ana L. S. Fraiha, Brenda M. M. Rocha, Barbara C. Longo, Roselene Ecco, Guilherme C. Faria, Ronaldo Furtini, Safira R. M. Drumond, Renata P. A. Maranhão, Zélia I. P. Lobato, Maria Isabel M. C. Guedes, Raffaella B. C. Teixeira, Erica A. Costa

**Affiliations:** 1 Departamento de Virologia e Terapia Experimental, Instituto Aggeu Magalhães, Fundação Oswaldo Cruz, Recife 50670-420, Brazil or lgilfioctuz@gmail.com (L.H.V.G.G.); or tecamagalhaes@hotmail.com (T.M.); liviaolvr@gmail.com (L.V.O.); 2 Center for Vector-Borne Infectious Diseases, Microbiology, Immunology and Pathology Department, Colorado State University, Fort Collins, CO 80523-1685, USA; 3 Laboratorio de Pesquisa em Virologia Animal, Escola de Veterinária, Universidade Federal de Minas Gerais, Belo Horizonte 31270-901, Brazil; beatrizsenra.santos@gmail.com (B.S.A.S.S.); jaumlrc@gmail.com (J.L.R.C.); anasoaresfraiha@hotmail.com (A.L.S.F.); brendammrocha@hotmail.com (B.M.M.R.); barbaraalongo@gmail.com (B.C.L.); ziplobato@gmail.com (Z.I.P.L.); mariaisabel.guedes@gmail.com (M.I.M.C.G.); 4 Institute of Virology, Charité-Universitätsmedizin Berlin, 10117 Berlin, Germany; edmilson.oliveira@charite.de; 5 Setor de Patologia, Escola de Veterinária, Universidade Federal de Minas Gerais, Belo Horizonte 31270-901, Brazil; eccoro.ufmg@gmail.com; 6 Laboratório de Saúde Animal, Instituto Mineiro de Agropecuária, Belo Horizonte 30110-005, Brazil; lsa@ima.mg.gov.br (G.C.F.); ronaldoanas@ig.com.br (R.F.); srmdrumond@hotmail.com (S.R.M.D.); 7 Clínica de Equinos, Hospital Veterinário, Universidade Federal de Minas Gerais, Belo Horizonte 31310-250, Brazil; rpamaranhao@yahoo.com; 8 Departamento de Veterinária, Universidade Federal de Viçosa, Viçosa 36571-000, Brazil; teixeiraraffa@gmail.com

**Keywords:** arbovirus, mosquito, Aedes, Culex, horse, neurological disease

## Abstract

Madariaga virus (MADV) is a member of the eastern equine encephalitis virus (EEEV) complex that circulates in Central and South America. It is a zoonotic, mosquito-borne pathogen, belonging to the family *Togaviridae*. Disturbances in the natural transmission cycle of this virus result in outbreaks in equines and humans, leading to high case fatality in the former and acute febrile illness or neurological disease in the latter. Although a considerable amount of knowledge exists on the eco-epidemiology of North American EEEV strains, little is known about MADV. In Brazil, the most recent isolations of MADV occurred in 2009 in the States of Paraíba and Ceará, northeast Brazil. Because of that, health authorities have recommended vaccination of animals in these regions. However, in 2019 an equine encephalitis outbreak was reported in a municipality in Ceará. Here, we present the isolation of MADV from two horses that died in this outbreak. The full-length genome of these viruses was sequenced, and phylogenetic analyses performed. Pathological findings from postmortem examination are also discussed. We conclude that MADV is actively circulating in northeast Brazil despite vaccination programs, and call attention to this arbovirus that likely represents an emerging pathogen in Latin America.

## 1. Introduction

Eastern equine encephalitis virus (EEEV), western equine encephalitis virus (WEEV), and Venezuelan equine encephalitis virus (VEEV) are neurotropic, mosquito-borne alphaviruses of zoonotic and public health importance circulating in the Americas [1]. They belong to the genus *Alphavirus*, family *Togaviridae*, and have a positive-sense, single-stranded, nonsegmented RNA genome of approximately 11.7 Kb [1]. EEEV is an important pathogen within this group as it can be deadly or cause severe neurological sequelae in animals and humans [2,3]. Initial antigenic features have led to the differentiation of EEEV isolates into the North American (NA EEEV) and South American (SA EEEV) lineages [4]. Further phylogenetic and antigenic analyses led to the classification of EEEV into four main lineages (I–IV), with isolates from NA and some countries of the Caribbean clustering into lineage I, and isolates from Central and South America clustering into lineages II–IV [5].

The primary transmission cycle of EEEV leading to endemicity is enzootic; however, infections of animal species that are not the main reservoirs and of humans occur when factors disturbing the natural transmission cycles result in spillover events. Despite the important fact that EEEV infection can result in severe disease or death in equines and humans, these are considered dead-end hosts and, thus, do not contribute to EEEV transmission. In its natural cycle, NA EEEV is transmitted among passerine birds by *Culiseta melanura* mosquitoes [6,7]; however, it is unclear what vectors and reservoirs are responsible for SA EEEV maintenance and amplification in nature.

Outbreaks of encephalitis and death in equines caused by SA EEEV strains have been reported in Central and South American countries in the past decades [8,9,10,11] but pathogenicity to humans appears to be considerably lower than that of the NA strains. Nevertheless, a human encephalitis outbreak of SA EEEV in 2010 in Panama raised questions of whether some SA strains are becoming more pathogenic to humans [2]. Due to genetic divergence, distinct evolutionary patterns, and differences in eco-epidemiology and pathogenesis among strains of lineage I (NA) and those of lineages II–IV (SA), in 2010 it was proposed that strains from lineages II–IV should be considered a distinct species within the EEEV complex. The new proposed species received the name Madariaga virus (MADV) [12].

In Brazil, impactful equine epizootics caused by EEEV have been reported in all five Brazilian regions, with the first EEEV isolations having occurred in the 1940s, followed by several reports in the next decades [10,13,14,15,16,17,18,19,20,21,22,23]. Phylogenetic analyses show that most Brazilian isolates cluster within lineages II and III [12,21,22,23].

**Figure 1:**
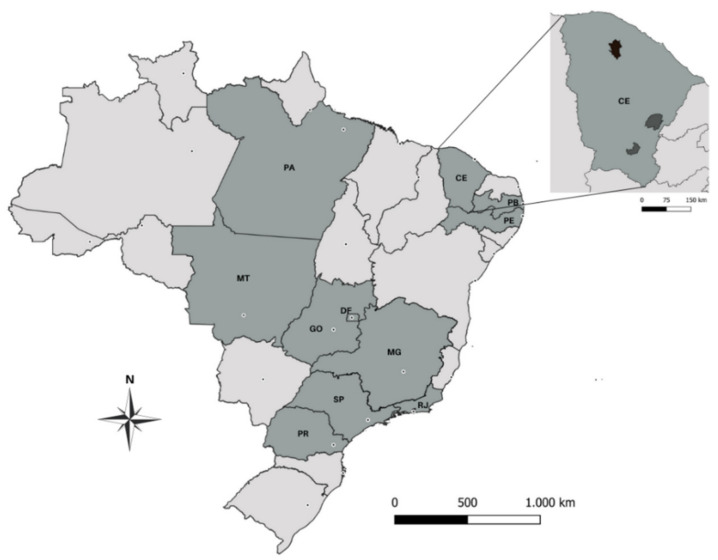
Map of Brazil showing (in dark gray) the Brazilian States where epizootics of South American eastern equine encephalitis (SA EEEV), or Madariaga virus (MADV), have occurred in equines. PA: Pará; MT: Mato Grosso; GO: Goiás; DF: Distrito Federal; MG: Minas Gerais; SP: São Paulo; RJ: Rio de Janeiro; PR: Paraná; CE: Ceará; PB: Paraíba; PE: Pernambuco. The State of Ceará is displayed separately with the municipalities where MADV infections in horses were detected in 2009 (darker gray) [29] and in the present study (black).

Circulation of SA EEEV in Brazil has also been evidenced by serosurveys, which have been mostly performed during equine epizootics or in areas where equine encephalitis cases had been previously reported. An early serosurvey in 1940–41 in the State of Minas Gerais (southeast (SE) region) performed during an epizootic with high case-fatality rates showed a seropositivity of 44.4% among alive equines [24]. In a region historically affected by EEEV epizootics in the State of São Paulo (SE region), seropositivity in equines was 16% in 2004–2005 [25]. In 1993, in the State of Mato Grosso (midwest (MW) region) 6.4% among hundreds of surveyed healthy equines were seropositive for EEEV in an area with reports of equine encephalitis cases [26], while in 2007 47.7% of non-vaccinated equines were seropositive in the same region [27]. In the State of Paraná (south (S) region), 54.5% of equine samples collected throughout 4 years in the 1990s were seropositive [28]. In the north (N) region, 69% of seropositivity in equines was observed in the State of Pará in the 1960s during ongoing epizootics [15], and 21% were seropositive in 2002 in Rondônia in a survey involving all equines from one municipality [17]. Regions where the main equine epizootics occurred in Brazil are shown in Figure 1.

Virus isolation and serosurveys in other animals have also been carried out in Brazil to infer their susceptibility to EEEV infection and/or potential role as reservoirs in the virus’s transmission cycle. EEEV has been isolated from avian species [10,16], terrestrial mammals (e.g., rodents and marsupials) [10,16], and even reptiles [10]. Seropositivity for EEEV ranged from 0 to 10% in birds from the N region [10,15], and was 2.9% in wild boars from three regions (S, SE, and MW) [30], 1% in a sloth species from the NE region [31], and 5% in opossum from the N region [15]. Sentinel animals have also led to virus isolation [10].

In humans, SA EEEV was isolated once from a person in the 1950s in NE Brazil [14], and detected molecularly in a person presenting with acute febrile illness in 2015–2016 in Mato Grosso (MW region) [21]. Serosurveys in humans are scarce but have shown the presence of EEEV antibodies in persons living in areas where equine epizootics have occurred and a seroprevalence range of 2.2–25% among surveyed populations from different regions [10,32,33], indicating that people living in areas where the virus circulates are at risk of infection.

Here, we present the isolation and full-length genome sequencing of SE EEEV (MADV) isolated in 2019 from two horses that died with encephalitis in the State of Ceará, NE Brazil. We then discuss their phylogeny, pathological findings, and the relevance of MADV active circulation in Brazil.

## 2. Results

### 2.1. Necropsy and Histopathology

Diffuse hyperemia was the only central nervous system (CNS) gross lesion observed in the two horses upon necropsy. One cerebrum section of animal 164 was examined by histopathology and no lesions were observed. Examination of two cerebrum sections of horse 165 revealed the following histopathologic findings: (1) moderate lymphoplasmacytic encephalitis was present mainly in the cerebral cortex (gray matter) and with a lesser extent in the white matter; (2) some vessels had swollen endothelium surrounded by one or three layers of lymphocytes and plasma cells; (3) nonsuppurative perivascular inflammation was seen in multifocal areas of the leptomeninges; (4) glial reaction was characterized by multiple areas with microglial proliferation in the neuroparenchyma. Microscopic changes as described above for horse 165 were indicative of viral infection.

### 2.2. Plaque Assay

**Figure 2:**
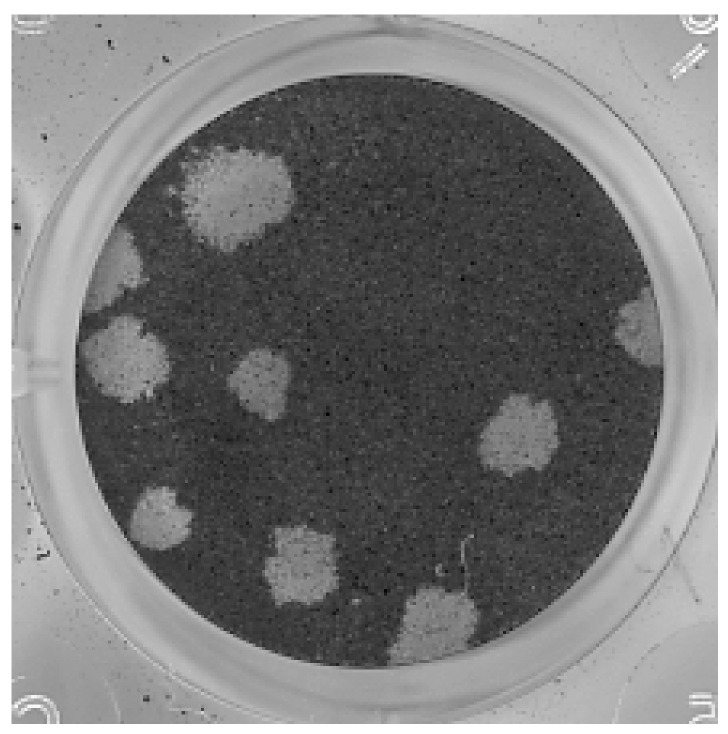
Plaque assay with samples 164 and 165 passage 5 (P5) was performed in Vero cells. Plaques were similar between the samples and measured 2–3 mm in diameter. The figure shows dilution 10^−5^ of sample 164.

Titers of 164 and 165 P5 were 1.5 × 10^8^ and 1 × 10^8^ PFU/mL, respectively. Plaques were similar in both samples and measured approximately 2–3 mm in diameter (Figure 2).

### 2.3. Flavivirus, Alphavirus, and Equine Encephalitis Virus Molecular Assays

**Figure 3:**
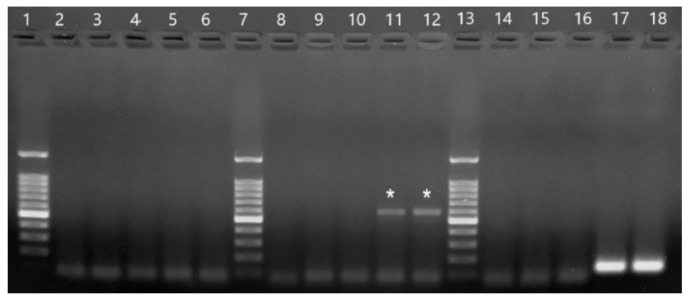
Products from primer-specific equine encephalitis virus PCRs. 2–6: PCR with WEEV primers. 8–12: PCR with VEEV primers. 14–18: PCR with EEEV primers. 1, 7, and 13:100 bp ladder (Promega). 2, 8, and 14: H_2_O. 3, 9, and 15: CHIKV PB302. 5, 11, and 17: sample 164. 6, 12, and 18: sample 165. The stronger band in the ladder is 500 bp and the lowest band is 100 bp. Wells 11 and 12 show the non-specific amplified fragments of 164 and 165 (marked with asterisks) with the VEEV forward primer and the genus-specific reverse alphavirus primer. Wells 17 and 18 show the specific EEEV band of 124 bp for samples 164 and 165, respectively.

Samples 164 and 165 were negative in the genus-specific flavivirus RT-PCR (no fragments were amplified). In the genus-specific alphavirus RT-PCR, a strong 434-bp fragment (expected band size) was observed in both samples. In the specific equine encephalitis virus PCRs, both samples tested negative for WEEV, positive for VEEV (weak band of approximately 600 bp; not the expected size), and positive for EEEV (strong band of 124 bp; expected size). The controls worked properly in all PCRs. Amplified fragments in the equine encephalitis virus PCRs are shown in Figure 3.

### 2.4. Herpesvirus PCR

Except for the positive control (HSV-1), no sample yielded visible bands in the universal herpesvirus PCR.

### 2.5. Sequencing Results and Phylogenetic Analysis

The first sequenced fragments (143–600 Kb) from 164 and 165 led to the identification of EEEV/MADV through BLASTn. The full-length genome was successfully sequenced through the Sanger method after several rounds of RT-PCRs. The genomes have 11,605 nucleotides and contain two ORFs. The first ORF has 7416 nucleotides and encodes the nonstructural proteins (nsP1–nsP4), while the second ORF is 3729 nucleotides long and encodes the structural polyprotein (C, E2, E3, 6K, E1). A conserved “leaky” stop codon was identified at position 5614. There was 99.9% of nucleotide identity between the genomes of 164 and 165, with only 9 mismatches. The sequences of samples 164 and 165 were submitted to GenBank (Accession numbers MZ389693 and MZ389692, respectively).

**Figure 4:**
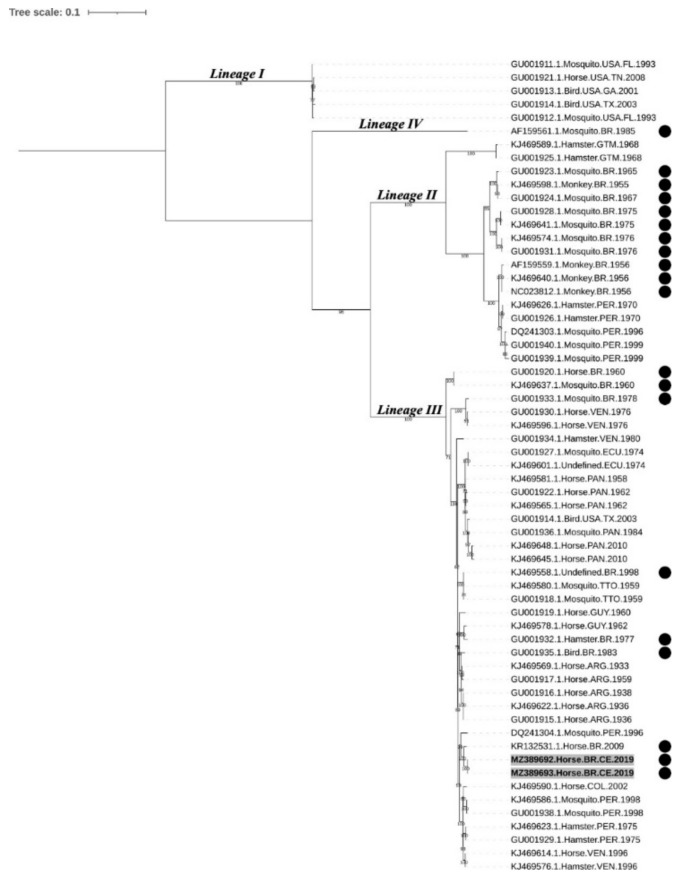
Nucleotide maximum likelihood phylogenetic analysis of 61 strains of EEEV based on the structural polyprotein open reading frame. Sequences aligned in different clusters refer to North American EEEV lineage (NA EEEV lineage I) and MADV lineages (II, III, and IV). Samples highlighted in bold and with a gray background indicate the sequenced samples of this study. Samples with a black circle were isolated in Brazil. Ultrafast bootstrap values were determined in 5000 replications; only bootstrap values ≥70% are shown. EEEV: eastern equine encephalitis virus; MADV: Madariaga virus.

The maximum likelihood phylogenetic tree (Figure 4) supports the previously defined EEEV lineages [5,12,29]. Sample sequences from this study (MADV#164 and MADV#165) shared similarities of 94.6% to 99% with other Brazilian sequences from lineage III, with higher values shared with KR132531.1 (99.0%), isolated from a horse in Paraíba, NE Brazil, in 2009, followed by GU001935.1, isolated from a bird in 1983 (97.4%). Sequence DQ241304 isolated from a mosquito in 1996 in Peru (also clustered within lineage III) shared 97.9% of similarity with sequences from this study. When compared to Brazilian sequences within lineages II and IV, MADV#164 and MADV#165 shared a similarity ranging from 81.6–82.5% and 80.4, respectively. The similarity between the sequences were estimated by the p-distance method [34]. The p-distance of Brazilian isolates is shown in Supplementary Table S3.

## 3. Discussion

The equine encephalitis outbreak reported here leading to the isolation of MADV occurred in 2019 in the State of Ceará, where an outbreak had been reported in 2009 [29]. In addition, SA EEEV outbreaks have also been reported in other regions of NE Brazil [19,29,35], indicating that the virus may be endemic in the NE region as it may be in other parts of the country. Importantly, even though EEEV vaccination in horses was carried out in Ceará after the 2009 outbreak and no equine encephalitis cases were reported in the following years, the outbreak in 2019 indicates that broad vaccination programs in animals are not being achieved in the region.

Endemicity of MADV in Brazil is truly relevant for animal and human health considering that infection with this pathogen can result in high morbidity and mortality in equines [24,29] and has the potential to cause disease in humans [2,21,36,37]. As for human health, although SA EEEV/MADV strains have been considered less pathogenic than NA strains for humans, MADV infection was confirmed in persons with severe neurological disease during an encephalitis outbreak in Panama in 2010 [2]. Changes in the transmission patterns and virulence are common among arboviruses, which have the ability to adapt to new hosts, vectors, and ecological niches.

The clinical signs observed in the two horses (ataxia, paresis, circling, knuckling, and recumbency) that ultimately died, appear to be common in cases of eastern equine encephalitis [20,29,38]. Other acute signs of EEEV infection that were not observed here include fever, abnormal gait, blindness, head-pressing, paralysis, severe depression, and convulsions [29,38,39,40,41]. In Brazil, any neurological disease in herbivores must be notified to the animal health regulatory agency (Ministério da Agricultura, Pecuária e Abastecimento: MAPA), as part of the national surveillance program for rabies. In these cases, CNS samples from animals should be collected and processed following MAPA’s protocol so that appropriate tests can be performed, and conclusions drawn. For histopathology, it is recommended that CNS samples should be fixed immediately upon collection. There are also specifications on the types of CNS tissues that must be collected as infection with different pathogens leads to pathology in distinct CNS areas [42]. For instance, brain lesions caused by EEEV are generally described in the cerebral cortex, hypothalamus, thalamus, and to a lesser extent, in the caudal portion of thalamus, other portions of brainstem and cerebellum [43,44]. The cervical portion of the spinal cord may also be affected [44]. Unfortunately, the reality is that in the field a significant number of neurological diseases in animals are not notified and, when they are, in most cases samples are not collected and prepared appropriately, leading to inconclusive diagnosis. Histopathology is critical to guide further tests towards a conclusive diagnosis of animal neurological disease, i.e., histopathologic analysis may indicate the etiologic agent causing the disease (e.g., viruses, protozoans, or bacteria) so that specific tests can be performed.

Here, lesions in horse 165 were more pronounced in the cerebral cortex; however, other regions of the CNS were not sampled for histopathology and more conclusions could not be reached. Importantly, the microscopic lesions in this animal, including the nonsuppurative encephalitis, were indicative of viral infection. Microglial proliferation and nonsuppurative inflammation, especially in the gray matter, can occur in a more advanced stage of this type of viral infection [43]. Neutrophil infiltration in the neuroparenchyma, sometimes around neurons, is a reaction seen in an earlier stage of the disease or in a peracute lethal course of the disease [43], and was not observed in this horse. Nevertheless, in the present study CNS samples were received frozen in the laboratory and then fixed (as opposed to being fixed immediately upon collection), which resulted in poor-quality processed tissues. Although the histopathology analysis led to some important observations, it is likely that the poor quality of the material due to improper conservation prevented additional conclusions. Moreover, the lack of abnormal histologic features in samples from horse 164 might have been due to improper sampling.

As the samples tested negative for rabies and because histology observations were indicative of viral infection, virus isolation and molecular diagnostic tests were carried out and led to the final diagnosis. We call attention to the fact that the viruses had to be amplified in tissue culture and the published flavivirus and alphavirus genus-specific RT-PCRs protocols had to be optimized to work properly (based on the positive controls). This is important as the low amount of virus in the original samples and the lack of optimizations in molecular assays can lead to false negative results. Issues concerning sample collection/processing and molecular assays must be considered as these are directly associated with the success of surveillance and control programs of zoonotic diseases.

The phylogenetic analyses showed that MADV#164 MADV#165 clustered within lineage III. Although strains from lineages II and IV (especially the former) have been detected in Brazil, the most recent studies have identified lineage III strains [21,22,23], suggesting this is currently the predominant lineage circulating in the country.

According to the evolutionary profile of MADV and to comparisons with NA EEEV and the closely related VEEV, it has been hypothesized that although some bird species are susceptible to MADV infection, the main amplifying reservoirs may be ground-dwelling mammals. In Brazil, studies with SA EEEV/MADV in the N region have shown that several bird species are susceptible to infection, given that some species exhibited (low) seropositivity. However, in these studies the seropositivity rates and the number of avian species potentially susceptible to MADV were considerably lower compared to Saint-Louis encephalitis virus (SLEV) and WEEV, which are arboviruses that have avian hosts as the main amplifying reservoirs [10]. In addition, EEEV seropositivity in various mammal species indicates they may be potential hosts [30,31]. The MADV outbreak in Panama also shed some light on the types of reservoirs involved in the local transmission cycle of this virus as the animal with the highest seropositivity rate for MADV was the short-tailed cane mouse [45]. As for relevant vectors, although MADV has been isolated from *Aedes taeniorhynchus* [15] and other *Aedes* spp. [10] in Brazil, several isolations from *Culex* spp., especially from *Cx.* (Melanoconion) spp. indicate that species of this genus may be playing an important role in transmission. Interestingly, *Cx.* (Melanoconion) spp. from a region in Brazil where several MADV epizootics have occurred (Vale da Ribeira, State of São Paulo) exhibited the most eclectic feeding behavior compared to other mosquito species [46] and some level of domestication [47]—excellent features for a vector involved in epizootic transmission and spill over events of arboviruses.

## 4. Conclusions

MADV is a potential emerging pathogen in Latin America and the several knowledge gaps regarding its transmission cycle, pathogenicity in different hosts, and attack rates, among others, warrant further research at many levels. Surveillance programs should start considering MADV as an endemic zoonotic agent in Brazil and, according to reports on MADV detection in acute human febrile illnesses in Venezuela [36], Haiti [37], and Brazil [21], MADV should also be considered in the differential diagnosis of arboviruses causing acute febrile disease in human populations and not only in neurological disease.

## 5. Materials and Methods

### 5.1. Outbreak Data and Sample Processing

On 30 May 2019, cases of acute equine neurological disease were notified to the local health authorities in Irauçu, State of Ceará, NE Brazil. One out of twelve, and six out of twelve horses from two farms (farm 1 and farm 2) became sick and died within five days from the onset of clinical signs. One horse from each farm was necropsied for postmortem diagnosis. Both horses were 5-year-old crossbreeds of unknown gender that exhibited signs like ataxia, paresis, circling, and knuckling, that progressed to recumbency and death. Upon necropsy, CNS fragments were collected and sent to the designated laboratory. Horses were the only affected species on the farms. Previous vaccination history was unknown, except that the herd had not been vaccinated for rabies. Farm 1 is located at 03°46′8.7″ latitude and 39°47′51.1″ longitude, and farm 2 at 03°46′31″ latitude and 39°46′12″ longitude. The farms are 3.2 km apart from each other.

Fresh CNS samples received by the Animal Health Laboratory at the Animal Defense Agency in the State of Minas Gerais (Laboratório de Saúde Animal, Instituto Mineiro de Agropecuária: LSA-IMA) were processed and tested for rabies by the direct fluorescent antibody and mouse inoculation tests, for which they were negative. For histopathology analysis, one or two sections of the cerebral hemispheres were fixed in 10% formalin, processed by routine histologic methods, embedded in paraffin, sectioned at 5 µm, and stained with hematoxylin/eosin (HE), according to Luna [48].

Samples were then sent to the Laboratory of Animal Virology Research at the Veterinary School (Laboratório de Pesquisa em Virologia Animal, Escola de Veterinária) of Universidade Federal de Minas Gerais and the Virology Department at Instituto Aggeu Magalhães/Fundação Oswaldo Cruz for the experiments describe hereafter.

### 5.2. Virus Isolation and Plaque Assay

Frozen CNS fragments from the two horses (horses’ ID: 164, farm 2; 165, farm 1) were homogenized using mortar and pestle and diluted 1:20 (w/v) in phosphate buffered saline (PBS) containing antibiotics and antifungal. The suspensions were vortexed for 15 sec, kept at 4 °C overnight, and centrifuged at 4000× *g* for 10 min at 4 °C. The supernatant was then collected and filtered through a 0.22 μm filter. Virus isolation was performed according to the protocol described by Pinheiro et al. [49], with modifications. Briefly, an aliquot of the samples was inoculated into 60–70% confluent Vero cells (ATCC-CCL-81) for a 1 h incubation (adsorption) at 37 °C (with gentle rocking at every 15 min) in a 5% CO_2_ incubator. The inoculum was removed, and medium was added into the flasks, which were incubated for approximately 3–5 days until most cells exhibited a cytopathic effect. The flask content was then harvested, centrifuged at 1000× *g* for 5 min, and the supernatant stored at −80 °C. The negative control consisted of cells inoculated with PBS. Four more rounds of virus amplification were performed the same way but instead of using homogenized CNS tissue, an aliquot of the previous passage was used for cell inoculation.

Plaque characterization and virus titer of 164 and 165 passage 5 (P5) were determined through plaque assays in Vero cells [50]. Briefly, cells were seeded into 24-well plates and 24 h later inoculated with 10-fold serial dilutions of the samples (100 µL of 10^−1^ to 10^−6^ dilutions after medium removal). After a 1 h adsorption at 37 °C/5% CO_2_ the inoculum was removed, and semi-solid medium (1.5% carboxymethyl cellulose in 2× tissue culture medium) was added to the wells. Cells were incubated at 37 °C/5% CO_2_ for 6 days and then fixed in 4% formalin after medium removal. Plaques were stained with 1% crystal violet (Sigma-Aldrich; St. Louis, United States), washed and counted visually for titer calculation, which was expressed as plaque forming units per mL (PFU/mL).

### 5.3. Molecular Assays

#### 5.3.1. RNA Extraction

A 250 uL aliquot of 164 and 165 P5 frozen stocks was used for RNA extraction using TRIzol Reagent (Invitrogen; Waltham, United States), according to the manufacturer’s instructions. After the 75% ethanol wash, the precipitate was air-dried and dissolved in 30 uL of Ultra-Pure H_2_O. Samples were stored at −80 °C until further analysis. In addition, RNA of Yellow Fever virus (YFV) strain 17DD and Chikungunya virus (CHIKV) strain PB302 that had been cultivated in Vero cells was extracted using the same protocol.

#### 5.3.2. Genus-Specific Flavivirus and Alphavirus RT-PCRs

A genus-specific nested RT-PCR targeting the NS5 flavivirus gene was optimized based on the method described by Sanchez-Seco et al. [51]. In the Sanchez-Seco strategy, a reverse-transcription (RT) reaction is performed, followed by a PCR to amplify a 1385 bp fragment of the NS5 gene, and a final nested-PCR to amplify a 143 bp portion of the first PCR product. Here, a 20-uL RT reaction was performed using the SCRIPT cDNA synthesis kit (Jena Bioscience; Jena, Germany) following the manufacturer’s instructions, and 8 µL of viral RNA. Prior to the RT reaction, RNA was mixed with the first-round PCR reverse primer Flavi1 (−) and incubated for 1 min at 70 °C for denaturation and primer annealing. The RT was then performed at 45 °C for 2 h, followed by a reverse transcriptase inactivation at 70 °C for 10 min. The 50-µL first-round PCR was performed with primers Flavi1 (+)/Flavi1 (−), KlenTaq DNA polymerase (Cellco Biotec; São Carlos, Brazil), and 5 uL of the RT product under the following cycling conditions: 95 °C for 2 min (initial denaturation); 40 cycles of 95 °C for 30 sec (denaturation), 47 °C for 30 sec (annealing), and 68 °C for 1 min 15 sec (extension); 68 °C for 2 min (final extension). The 50-µL nested-PCR was performed with primers Flavi2 (+)/Flavi2 (−), KlenTaq, and 1 µL of the first-round PCR product under the same cycling conditions as the first-round PCR, except that the extension time was 30 sec and there were 35 cycles. The positive control for the flavivirus RT-PCR was YFV 17DD RNA, and the negative controls were H_2_O and CHIKV PB302 RNA.

A genus-specific alphavirus RT-PCR was optimized based on the strategy reported by Pfeffer et al. [52]. In the Pfeffer strategy, primers M2W (+)/cM3W (−) are used to amplify a 434 bp region of the nsP1 gene from alphaviruses. The RT reaction was performed using the reverse primer cM3W (−) the same way as described above. PCR cycling conditions were the same as those described for the nested flavivirus PCR, except that the annealing temperature was 50 °C. The positive control for the alphavirus RT-PCR was CHIKV PB302 RNA, and the negative controls were H_2_O and YFV 17DD RNA.

#### 5.3.3. Equine Encephalitis Virus PCRs

The product from the RT reaction with the genus-specific alphavirus reverse primer (described above) was used in a modified nested, primer-specific PCR for WEEV, VEEV, and EEEV, following a previously described strategy [53]. For that, 5 uL of the RT product was used in singleplex PCRs with WEEV-, VEEV-, or EEEV-specific forward primers (nWEE, nVEE, and nEEE, respectively) and the genus-specific alphavirus reverse primer cM3W (−). PCR conditions were the same as the alphavirus PCR, except that the annealing temperature was 52 °C. The expected amplicon size for WEEV, VEEV, or EEEV was 208, 400, and 124 bp, respectively. The negative controls in these RT-PCRs were H_2_O and CHIKV PB302; positive controls were not available.

#### 5.3.4. DNA Extraction and Herpesvirus PCRs

A nested-PCR to detect herpesviruses was performed with samples 164 and 165. For that, DNA was extracted using 500 µL of 164 and 165 frozen stocks using the phenol:chloroform extraction protocol described by ThermoFisher (Waltham, United States) [54], except that glycogen was replaced by linear acrylamide. DNA pellets were resuspended in 50 µL of ultra-pure H_2_O. Herpesvirus PCR was then performed according to VanDevanter [55]. In the first reaction, 2 µL of DNA were used in a 50-µL reaction using KlenTaq, and PCR conditions were the following: 95 °C for 2 min (initial denaturation); 40 cycles of 95 °C for 30 sec (denaturation), 46 °C for 30 sec (annealing), and 68 °C for 1 min (extension); 68 °C for 2 min (final extension). In the nested-PCR, 5 µL of the first PCR was used in a 50-µL reaction using KlenTaq, and PCR conditions were the same, except that the extension was allowed to occur for 30 sec. The expected band size after the nested-PCR was 215–315 bp. The positive control for this assay was herpesvirus simplex 1 (HSV-1) from cell culture, and the negative control was H_2_O.

### 5.4. Sequencing

The amplicons of samples 164 and 165 from the genus-specific alphavirus PCR and the equine encephalitis virus PCRs were sequenced by the chain termination (Sanger) method with the same primers used in the respective PCRs. Nucleotide sequence similarity searching using the Basic Local Alignment Search Tool (BLASTn) (http://blast.ncbi.nlm.nih.gov, accessed on 26 November 2020) showed high similarity of the amplified material with MADV sequences deposited in the database. Next, whole genome sequencing of both samples was achieved through Sanger sequencing. Initially, primers were designed based on a MADV sequence retrieved from GenBank (accession number KR132531.1) and then sequences obtained from the first-round sequencing were used for further primer design to be used in RT and PCR. RT reactions were first performed with the reverse primers as described above (except that 4 µL of RNA were used and RT reaction occurred for 1 h), and PCR was then performed as described above for the genus-specific alphavirus PCR, except that the annealing temperature varied from 52 to 65 °C and the extension time was adapted to the size of each fragment to be amplified (1 min/Kb). Primers were designed with the “A plasmid Editor-ApE” program. The sequences of the primers designed to obtain the full-length genome sequencing of the samples are shown in Supplementary Table S1. Alignments and genome assembly was performed with ApE. In all cases, before being sent for sequencing PCR products were run in 1% agarose gel and the bands of interest cut and purified with the Zymoclean Large Fragment DNA Recovery Kit (Zymo Research; Irvine, United States).

### 5.5. Phylogenetic Analysis

Sequences were aligned with reference sequences obtained from GenBank (Supplementary Table S2) using MAFFT v7.471 [56], global pair method, and adjust direction option. The phylogeny, based on the structural polyprotein open reading frame (ORF) of EEEV and MADV, was estimated by maximum likelihood, using IQ-TREE 2 [57], where the best nucleotide substitution model selected by the program was GTR+F+I+G4 [58]. Branch support was estimated using the ultrafast bootstrap (UFBoot) method with 5000 bootstrap replicates. The tree image was generated with the ITOL online tool [59]. The final tree was rooted at midpoint root. The pairwise distance between sequences were estimated by the p-distance method under 1000 bootstrap replications using Mega 7.0.26. software [34].

## Data Availability

The full genome sequences of samples 164 and 165 were submitted to GenBank (Accession numbers MZ389693 and MZ389692, respectively).

## Supplementary Materials

The following are available online at https://www.mdpi.com/article/10.3390/pathogens10080983/s1, Supplementary Table S1: Madariaga virus (MADV) primers used for reverse-transcription (RT)-PCRs and sequencing of samples 164 and 165, Supplementary Table S2: Reference sequences used in this study with the respective GenBank Accession numbers, and Supplementary Figure S1: P-distance analysis of Brazilian sequences from lineages II, III, and IV, based on the structural polyprotein open reading frame.

## References

[1] Zacks M.A., Paessler S. (2010). Encephalitic alphaviruses. Vet. Microbiol..

[2] Carrera J.P., Forrester N., Wang E., Vittor A.Y., Haddow A.D., Lopez-Verges S., Abadia I., Castano E., Sosa N., Baez C. (2013). Eastern equine encephalitis in Latin America. N. Engl. J. Med..

[3] Morens D.M., Folkers G.K., Fauci A.S. (2019). Eastern Equine Encephalitis Virus - Another Emergent Arbovirus in the United States. N. Engl. J. Med..

[4] Casals J. (1964). Antigenic Variants of Eastern Equine Encephalitis Virus. J. Exp. Med..

[5] Brault A.C., Powers A.M., Chavez C.L., Lopez R.N., Cachon M.F., Gutierrez L.F., Kang W., Tesh R.B., Shope R.E., Weaver S.C. (1999). Genetic and antigenic diversity among eastern equine encephalitis viruses from North, Central, and South America. Am. J. Trop. Med. Hyg..

[6] Molaei G., Thomas M.C., Muller T., Medlock J., Shepard J.J., Armstrong P.M., Andreadis T.G. (2016). Dynamics of Vector-Host Interactions in Avian Communities in Four Eastern Equine Encephalitis Virus Foci in the Northeastern U.S. PLoS Negl. Trop. Dis..

[7] Armstrong P.M., Andreadis T.G. (2010). Eastern equine encephalitis virus in mosquitoes and their role as bridge vectors. Emerg. Infect. Dis.

[8] Dietz W.H., Galindo P., Johnson K.M. (1980). Eastern equine encephalomyelitis in Panama: The epidemiology of the 1973 epizootic. Am. J. Trop. Med. Hyg..

[9] Sabattini M.S., Daffner J.F., Monath T.P., Bianchi T.I., Cropp C.B., Mitchell C.J., Aviles G. (1991). Localized eastern equine encephalitis in Santiago del Estero Province, Argentina, without human infection. Medicina.

[10] Vasconcelos P.F.C., Travassos da Rosa J.F.S., Travassos da Rosa A.P.A., Degallier N., Pinheiro F.P., Sá Filho G.C. (1991). Epidemiologia das encefalites por arbovírus na amazônia brasileira. Rev. Inst. Med. Trop. S. Paulo.

[11] Medina G., Gleiser C.A., Mackenzie R.B. (1965). Outbreak of equine encephalomyelitis in the Republic of Panama. Bol. Oficina Sanit Panam..

[12] Arrigo N.C., Adams A.P., Weaver S.C. (2010). Evolutionary patterns of eastern equine encephalitis virus in North versus South America suggest ecological differences and taxonomic revision. J. Virol..

[13] Alice F.J. (1951). Encefalomielite eqüina na Bahia: Estudo de três amostras isoladas. Rev. Brasil. Biol..

[14] Alice F.J. (1956). Infecção humana pelo vírus “leste” da encefalite equina. Bol. Inst. Biol. Bahia.

[15] Causey O.R., Shope R.E., Sutmoller P., Laemmert H. (1962). Epizootic eastern equine encephalitis in the Bragança region of Pará, Brazil. Rev. Serv. Esp. Saúde Pública.

[16] Lopes O.S., Sacchetta L.A. (1974). Epidemiological studies on Eastern Equine Encephalitis virus in São Paulo, Brazil. Rev. Inst. Med. Trop. São Paulo.

[17] Aguiar D.M., Cavalcante G.T., Lara M.C.C.S.H., Villalobos E.M.C., Cunha E.M.S., Okuda L.H., Stefano E., Nassar A.F.C., Souza G.O., Vasconcellos S.A. (2008). Prevalência de anticorpos contra agentes virais e bacterianos em eqüídeos do Município de Monte Negro, Rondônia, Amazônia Ocidental Brasileira. Braz. J. Vet. Res. Anim. Sci..

[18] Carneiro V., Cunha R. (1943). Estudos sobre a encefalomielite infecciosa dos equídeos no Brasil. Arch. Inst. Biol..

[19] Cunha R. (1945). Estudos sôbre uma amostra de vírus da encefalomielite equina isolada de material proveniente de Recife. Bol. Soc. Bras. Med. Vet..

[20] Campos K.F., Oliveira C.H.S., Reis A.B., Yamasaki E.M., Brito M.F., Andrade S.J.T., Duarte M.D., Barbosa J.D. (2013). Surto de encefalomielite equina Leste na Ilha de Marajó, Pará. Pesq. Vet. Bras..

[21] Costa M.C.S., Siqueira Maia L.M., Costa de Souza V., Gonzaga A.M., Correa de Azevedo V., Ramos Martins L., Chavez Pavoni J.H., Gomes Naveca F., Dezengrini Slhessarenko R. (2019). Arbovirus investigation in patients from Mato Grosso during Zika and Chikungunya virus introdution in Brazil, 2015-2016. Acta Trop..

[22] Oliveira R.N., Iamamoto K., Silva M.L., Achkar S.M., Castilho J.G., Ono E.D., Lobo R.S., Brandao P.E., Carnieli P., Carrieri M.L. (2014). Eastern equine encephalitis cases among horses in Brazil between 2005 and 2009. Arch. Virol..

[23] Silva M.L.C.R., Auguste A.J., Terzian A.C.B., Vedovello D., Riet-Correa F., Macario V.M.K., Mourao M.P.G., Ullmann L.S., Araujo J.P., Weaver S.C. (2017). Isolation and Characterization of Madariaga Virus from a Horse in Paraiba State, Brazil. Transbound Emerg. Dis..

[24] Lennette E.H., Fox J.P. (1943). Anticorpos neutralizantes para a amostra leste do vírus de encefalomielite equina em equídeos no Brasil. Mem. Inst. Oswaldo Cruz.

[25] Cunha E.M.S., Villalobos E.M.C., Nassar A.F.C., Lara M.C.C.S.H., Peres N.F., Palazzo J.P.C., Silva A., De Stefano E., Pino F.A. (2009). Prevalência de anticorpos contra agentes virais em equídeos no sul do estado de São Paulo. Arq. Inst. Biol. São Paulo.

[26] Iversson L.B., Silva R.A., Travassos da Rosa A.P., Barros V.L.R.S. (1993). Circulation of Eastern Equine Encephalitis, Western Equine Encephalitis, Ilheus, Maguari and Tacaiuma viruses in equines of the Brazilian pantanal, South America. Rev. Inst. Med. Trop. São Paulo.

[27] Pauvolid-Correa A., Tavares F.N., Costa E.V., Burlandy F.M., Murta M., Pellegrin A.O., Nogueira M.F., Silva E.E. (2010). Serologic evidence of the recent circulation of Saint Louis encephalitis virus and high prevalence of equine encephalitis viruses in horses in the Nhecolandia sub-region in South Pantanal, Central-West Brazil. Mem. Inst. Oswaldo Cruz.

[28] Fernandez Z., Richartz R., Travassos Da Rosa A., Soccol V.T. (2000). Identificação do vírus causador de encefalomielite equina, Paraná, Brasil. Rev. Saúde Pública.

[29] Silva M.L., Galiza G.J., Dantas A.F., Oliveira R.N., Iamamoto K., Achkar S.M., Riet-Correa F. (2011). Outbreaks of Eastern equine encephalitis in northeastern Brazil. J. Vet. Diagn. Invest..

[30] Kmetiuk L.B., Custodio de Souza Hunold Lara M.D.C., Monteforte Cassaro Villalobos E., de Barros Filho I.R., Martins C.M., Bach R.V.W., Pistori Machado F., Silva Pereira M., Cavalcante Lipinski L., Dos Santos A.P. (2020). Serosurvey of Eastern, Western, and Venezuelan Equine Encephalitis Viruses in Wild Boars (*Sus scrofa*), Hunting Dogs, and Hunters of Brazil. Vector Borne Zoonotic Dis..

[31] Catenacci L.S., Ferreira M., Martins L.C., De Vleeschouwer K.M., Cassano C.R., Oliveira L.C., Canale G., Deem S.L., Tello J.S., Parker P. (2018). Surveillance of Arboviruses in Primates and Sloths in the Atlantic Forest, Bahia, Brazil. Ecohealth.

[32] Bruno-Lobo M., Bruno-Lobo G., Travassos J. (1961). Estudos sôbre Arborvírus. II – Presença de anticorpos para certos vírus dos grupos A e B em soros de pessoas residentes no Rio de Janeiro. An. Microbiol..

[33] Romano-Lieber N.S., Iversson L.B. (2000). Inquérito soroepidemiológico para pesquisa de infecções por arbovírus em moradores de reserva ecológica. Rev. Saúde Pública.

[34] Kumar S., Stecher G., Tamura K. (2016). MEGA7: Molecular Evolutionary Genetics Analysis Version 7.0 for Bigger Datasets. Mol. Biol. Evol..

[35] Cunha R. (1943). Verificação de anticorpos para o vírus “Este” da encefalomielite equina em sôro de cavalos no nordeste brasileiro. Rev. Bras. Biol..

[36] Blohm G.M., Lednicky J.A., White S.K., Mavian C.N., Marquez M.C., Gonzalez-Garcia K.P., Salemi M., Morris J.G., Paniz-Mondolfi A.E. (2018). Madariaga Virus: Identification of a Lineage III Strain in a Venezuelan Child With Acute Undifferentiated Febrile Illness, in the Setting of a Possible Equine Epizootic. Clin. Infect. Dis..

[37] Lednicky J.A., White S.K., Mavian C.N., El Badry M.A., Telisma T., Salemi M., BA O.K., Beau De Rochars V.M., Morris J.G. (2019). Emergence of Madariaga virus as a cause of acute febrile illness in children, Haiti, 2015-2016. PLoS Negl. Trop. Dis..

[38] Sousa S.K.H., Sonne L., Sant’Ana F.J.F., Reis J.L. (2015). L. Encefalomielite equina do leste no Distrito Federal e entorno. Acta Sci. Vet..

[39] U.S. Department of Agriculture, Animal and Plant Health Inspection Service Equine Encephalitis (EEE/WEE/VEE). https://www.aphis.usda.gov/aphis/ourfocus/animalhealth/animal-disease-information/equine/eee-wee-vee/equine-encephalitis.

[40] Del Piero F., Wilkins P.A., Dubovi E.J., Biolatti B., Cantile C. (2001). Clinical, pathologic, immunohistochemical, and virologic findings of eastern equine encephalomyelitis in two horses. Vet. Pathol..

[41] Pimentel L.A., Oliveira D.M., Galiza G.J.N., Rego R.O., Dantas A.F.M., Riet-Correa F. (2009). Doenças do sistema nervoso central de equídeos no semi-árido. Pesq. Vet. Bras..

[42] Vandevelde M., Higgins R., Oevermann A. (2012). Veterinary Neuropathology: Essentials of Theory and Practice.

[43] Cantile C., Youssef S., Maxie M.G. (2016). Nervous system.

[44] Franklin R.P., Kinde H., Jay M.T., Kramer L.D., Green E.G., Chiles R.E., Ostlund E., Husted S., Smith J., Parker M.D. (2002). Eastern equine encephalomyelitis virus infection in a horse from California. Emerg. Infect. Dis..

[45] Vittor A.Y., Armien B., Gonzalez P., Carrera J.P., Dominguez C., Valderrama A., Glass G.E., Beltran D., Cisneros J., Wang E. (2016). Epidemiology of Emergent Madariaga Encephalitis in a Region with Endemic Venezuelan Equine Encephalitis: Initial Host Studies and Human Cross-Sectional Study in Darien, Panama. PLoS Negl. Trop. Dis..

[46] Forattini O.P., Gomes A.C., Natal D., Kakitani I., Marucci D. (1987). Preferências alimentares de mosquitos *Culicidae* no Vale do Ribeira, São Paulo, Brasil. Rev. Saúde Públ. S. Paulo.

[47] Forattini O.P., Gomes A.C., Kakitani I., Marucci D. (1991). Observações sobre domiciliação de mosquitos *Culex* (Melanoconion), em ambiente com acentuadas modificações antrópicas. Rev. Saúde Públ. S. Paulo.

[48] Luna L.G. (1968). Manual of Histologic Staining Methods of the Armed Forces Institute of Pathology.

[49] Pinheiro G.G., Rocha M.N., de Oliveira M.A., Moreira L.A., Andrade Filho J.D. (2019). Detection of Yellow Fever Virus in Sylvatic Mosquitoes during Disease Outbreaks of 2017(-)2018 in Minas Gerais State, Brazil. Insects.

[50] McAuley A.J., Beasley D.W., Colpitts T.M. (2016). Propagation and Titration of West Nile Virus on Vero Cells. West Nile Virus.

[51] Sanchez-Seco M.P., Rosario D., Domingo C., Hernandez L., Valdes K., Guzman M.G., Tenorio A. (2005). Generic RT-nested-PCR for detection of flaviviruses using degenerated primers and internal control followed by sequencing for specific identification. J. Virol. Methods.

[52] Pfeffer M., Proebster B., Kinney R.M., Kaaden O.R. (1997). Genus-specific detection of alphaviruses by a semi-nested reverse transcription-polymerase chain reaction. Am. J. Trop. Med. Hyg..

[53] Bronzoni R.V., Moreli M.L., Cruz A.C., Figueiredo L.T. (2004). Multiplex nested PCR for Brazilian Alphavirus diagnosis. Trans. R Soc. Trop. Med. Hyg..

[54] ThermoFisher Protocol—Phenol/Chloroform extraction. https://www.thermofisher.com/br/en/home/references/protocols/nucleic-acid-purification-and-analysis/dna-extraction-protocols/phenol-chloroform-extraction.html.

[55] VanDevanter D.R., Warrener P., Bennett L., Schultz E.R., Coulter S., Garber R.L., Rose T.M. (1996). Detection and analysis of diverse herpesviral species by consensus primer PCR. J. Clin. Microbiol..

[56] Katoh K., Standley D.M. (2013). MAFFT multiple sequence alignment software version 7: Improvements in performance and usability. Mol. Biol. Evol..

[57] Minh B.Q., Schmidt H.A., Chernomor O., Schrempf D., Woodhams M.D., von Haeseler A., Lanfear R. (2020). IQ-TREE 2: New Models and Efficient Methods for Phylogenetic Inference in the Genomic Era. Mol. Biol. Evol..

[58] Kalyaanamoorthy S., Minh B.Q., Wong T.K.F., von Haeseler A., Jermiin L.S. (2017). ModelFinder: Fast model selection for accurate phylogenetic estimates. Nat. Methods.

[59] Letunic I., Bork P. (2021). Interactive Tree Of Life (iTOL) v5: An online tool for phylogenetic tree display and annotation. Nucleic Acids Res..

