# Supplementary material for: Active Circulation of Madariaga Virus, a Member of the Eastern Equine Encephalitis Virus Complex, in Northeast Brazil
Source: Pathogens. 2021 Aug 3;10(8):983. doi: 10.3390/pathogens10080983 (PMC8400464; doi:10.3390/pathogens10080983)
Supplement: Supplementary file 1 [file pathogens-10-00983-s001.zip › pathogens-1278113-supplementary.pdf]

Supplementary Table 1 - S1: Madariaga virus (MADV) primers used for reverse-transcription (RT)-PCRs and sequencing of samples 164 and 165.

Primer name	5' - 3' sequence
MADV-1F	ATAGGGTATGGTGTAGAGGCAGCCAC
MADV-81F	CCCATACGTCAAGTCGCTGC
MADV-220F	CAGGTGATCCTGGACATTGGAAG
MADV-444F	GCACACAGACTCCACTTGTAGG
MADV-502F	GCAGTGCATGCACCTACCTC
MADV-978F	GAGCTGCAAGGTGACTGATACG
MADV-1009F	GAGAGGGTATCCTTTCCCGTGTG
MADV-1335F	CAAGAAACCAGGTACACAGACCATC
MADV-1453F	CTGGAAGAGAAGAAGAAGCC
MADV-1640F	GAGCGGGTAGTGTCGAAACG
MADV-1899F	GAGCGAAAGCGCGACTATTG
MADV-1993F	GAAGAGTACTACAAAGTCCTTAG
MADV-2503F	AACATGATGTGCCTCAAGGTG
MADV-2540F	TCTGTACCGAAGTGTACCACAAGAG
MADV-2630F	TGCGAACAGTGAATCCGTGC
MADV-2814F	CGCGGTCAGATACAAGGTCAAC
MADV-3066F	ACACGTATGCTGGGCGAAG
MADV-3157F	AAGGACGACAAAGCCTTTTCACC
MADV-3453F	TCGGCGACTTCCACATTCTC
MADV-3571F	GTCCCAGGCAAACGAGTTGAG
MADV-3804F	GGGTTATGGCACTGCTGATAGAG
MADV-3889F	AAATGTGCCTGGGAGAATACGGAG
MADV-4087F	AACGCGGCAAATAACAAAGGAC
MADV-4515F	GCTCGTGGAGGATGACAAACC
MADV-4604F	ATGAAGGTAAAGTACACTCCTAC
MADV-4787F	CCTCACCACCGCATACAATTCC
MADV-4902F	GCCTAAGTACAGAATAACAGGTGTGC
MADV-5085F	CATTCCAAGCCCACGCTGTTC
MADV-5312F	GCTCAATTACTCGCACGCCAG
MADV-5375F	TCACTGACATTTTCGAGAACTTC
MADV-5552F	CTGGCGTAGCAAGATCCATCTC
MADV-5692F	TGCAAACTCCAACACCCTATTCTG
MADV-5818F	AGCAGGTATCAATCGCGTAAAGTC
MADV-6091F	GACGGGGCATCCTGCTGTTTAG
MADV-6804F	CCTTGAGGATCTGGGTGTCGAC
MADV-7528F	TACAGAAACGATGTTTCCGTATCC
MADV-8187F	TGGACAACCGCGGTCGCGTGG
MADV-8973F	ACAAACGTGCAGATCAGGGAC
MADV-9616F	TACGTGTGCGGCCATTATTATGG
MADV-10242F	CGCAAATGAGCGAAGCGTAC
MADV-10311F	TGCACACAGGAACAGTGCAAG
MADV-10577F	GACCTGTACGCCAACACCAATC
MADV-10686F	GCGCACCATTGAACGACATTG
MADV-10787F	ATCCCTGATGCCGCGTTCACGAG
MADV-151R	CATTGTCGGTGACCTGCGTG
MADV-215R	GGGTCCACTTCGCTTTCGATG
MADV-390R	TGCAGCTTTGGAGGCTATGAAC
MADV-569R	CCTATCCAGTAGATGGTGCGCAC
MADV-676R	ACAGACCTATGTTCCTTGCCTC
MADV-1072R	TCCCTGTCATCTGATCGCACAGTG
MADV-1166R	GTGTTACGTTGTGTTCTTCCGTTGAC
MADV-1539R	CACTGCCTCCGCTTCTTCTTG
MADV-1615R	TGTCCGCTTCAACGGTTTCTC
MADV-1753R	TTTCGCTGTTAAGGACTGCCTG
MADV-1897R	GCGCTTGAAAGTCCGGGATTG
MADV-2108R	TCACCAACAAGGCACATTGG
MADV-2150R	AGACTTTCATACGCGAACTCGTG
MADV-2253R	ACGCTTCGTGACTGCACTC
MADV-2612R	AGTGTAGAAACTATTGCCGTGAC
MADV-2705R	ATGTCGTCTTTCAGCGGCTTC
MADV-2908R	AGACAATACGCTTCTCGGTACG
MADV-3045R	GCTATTGGCGGGAGTTTCTAACAC

MADV-3216R	AAAGAACCTGGTGCACAGGAAG
MADV-3770R	TCGACGGCTTTCCTGGTGAG
MADV-3794R	CAGGTGCCTCCTTTGTTCAAGTG
MADV-3982R	GCACGACACTTAGTTGATCTTG
MADV-4053R	TCCTCGAATGACTCTGTATGCTGG
MADV-4285R	CTTCTGACAGTTTCTGGTTGCCTTC
MADV-4336R	ACACCTTGTTGTACCGCTCC
MADV-4720R	GGCATATTTGCTCATTGGCTTCCTG
MADV-4867R	TCTTTGCCATTCGCAGTCTGTAG
MADV-4955R	ATGACTGGTTTGTTGCACTGGATC
MADV-5243R	CTAGCATGAACCTCTGCCTCTACG
MADV-5480R	TGAATGTCAGTCTCCGGCTCC
MADV-5513R	GGAACTGGTTTCTCCTCCG
MADV-5777R	AGCAATTTCTCACGTTCCAGGTC
MADV-5953R	ACACAGGGACAGGGTAATTGATC
MADV-6224R	TTCTGTAGGGTATTCTGTATAGG
MADV-6892R	TCCCTGTAGGTAAGTGAACAGATG
MADV-7584R	TTAACCGGCGCCATGGGTG
MADV-8313R	TCAGAACCTTCGGGAGTGTCC
MADV-9106R	ACAGTAATACTTCACTTGAGAGCC
MADV-9741R	ACTGCCAGCAGAATAGGTACTTG
MADV-10375R	AGCGGATCTCCAGCTGACGCTTC
MADV-10461R	AATGGAGTCCAGGCGGAAG
MADV-10892R	TGGCTATTCCTCCGAAATCAGAC
MADV-10990R	GCTGTCTGCCAACGTAACGTC
3'UTR-PolyA-Rev	TTTTTTTTTTTTTTTTTTTTTTGAAATATTAAAAACAAAATAAAACATAAAAGAAAAAC

Supplementary Table 2 - S2: Reference sequences used in this study with the respective GenBank Accession numbers.

GenBank Accession number	Host	Country	Collection year
KR132531	Horse	Brazil (BR)	2009
GU001929	Hamster	Peru (PER)	1975
GU001938	Mosquito	Peru (PER)	1998
KJ469586	Mosquito	Peru (PER)	1998
KJ469590	Horse	Colombia (COL)	2002
KJ469623	Hamster	Peru (PER)	1975
KJ469576	Hamster	Venezuela (VEN)	1996
KJ469614	Horse	Venezuela (VEN)	1996
DQ241304	Mosquito	Peru (PER)	1996
GU001915	Horse	Argentina (ARG)	1936
GU001916	Horse	Argentina (ARG)	1938
KJ469622	Horse	Argentina (ARG)	1936
GU001917	Horse	Argentina (ARG)	1959
KJ469569	Horse	Argentina (ARG)	1933
GU001935	Bird	Brazil (BR)	1983
GU001919	Horse	Guyana (GUY)	1960
GU001932	Hamster	Brazil (BR)	1977
KJ469578	Horse	Guyana (GUY)	1962
GU001918	Mosquito	Trinidad and Tobago (TTO)	1959
GU001934	Hamster	Venezuela (VEN)	1980
KJ469565	Horse	Panama (PAN)	1962
GU001922	Horse	Panama (PAN)	1962
KJ469581	Horse	Panama (PAN)	1958
KJ469601	Undefined	Ecuador (ECU)	1974
GU001927	Mosquito	Ecuador (ECU)	1974
GU001936	Mosquito	Panama (PAN)	1984
GU001937	Hamster	Colombia (COL)	1992
KJ469645	Horse	Panama (PAN)	2010
KJ469648	Horse	Panama (PAN)	2010
KJ469558	Undefined	Brazil (BR)	1998
KJ469580	Mosquito	Trinidad and Tobago (TTO)	1959
KJ469637	Mosquito	Brazil (BR)	1960
GU001920	Horse	Brazil (BR)	1960
GU001930	Horse	Venezuela (VEN)	1976
KJ469596	Horse	Venezuela (VEN)	1976
GU001933	Mosquito	Brazil (BR)	1978
KJ469641	Mosquito	Brazil (BR)	1975
GU001928	Mosquito	Brazil (BR)	1975
GU001931.	Mosquito	Brazil BR)	1976
KJ469574	Mosquito	Brazil (BR)	1976
GU001924	Mosquito	Brazil (BR)	1967
KJ469598	Monkey	Brazil (BR)	1955
GU001923	Mosquito	Brazil (BR)	1965
GU001939	Mosquito	Peru (PER)	1999
GU001940	Mosquito	Peru (PER)	1999
DQ241303	Mosquito	Peru (PER)	1996
GU001926	Hamster	Peru (PER)	1970
KJ469626	Hamster	Peru (PER)	1970
NC_023812	Monkey	Brazil (BR)	1956
KJ469640	Monkey	Brazil (BR)	1956
AF159559	Monkey	Brazil (BR)	1956
GU001925	Hamster	Guatemala (GTM)	1968
KJ469589	Hamster	Guatemala (GTM)	1968
AF159561	Mosquito	Brazil (BR)	1985
GU001913	Bird	United states (USA)	2001
GU001921	Horse	United states (USA)	2008
GU001911	Mosquito	United states (USA)	1993
GU001912	Mosquito	United states USA)	1993
GU001914	Bird	United states (USA)	2003

Supplementary Figure 1 - S1: P-distance analysis of Brazilian sequences from lineages II, III and IV, based on the structural polyprotein open reading frame.

		MADV164.BR.CE.2019	MADV165.BR.CE.2019
Lineage III	**MADV164.BR.CE.2019**	1	0.999
	**MADV165.BR.CE.2019**	0.999	1
	KR132531.1.Horse.BR.2009	0.990	0.990
	GU001935.1.Bird.BR.1983	0.974	0.974
	GU001932.1.Hamster.BR.1977	0.973	0.973
	KJ469558.1.Undefined.BR.1998	0.974	0.973
	KJ469637.1.Mosquito.BR.1960	0.955	0.955
	GU001920.1.Horse.BR.1960	0.955	0.955
	GU001933.1.Mosquito.BR.1978	0.947	0.946
Lineage II	KJ469641.1.Mosquito.BR.1975	0.816	0.816
	GU001928.1.Mosquito.BR.1975	0.816	0.816
	GU001931.1.Mosquito.BR.1976	0.824	0.824
	KJ469574.1.Mosquito.BR.1976	0.824	0.824
	GU001924.1.Mosquito.BR.1967	0.825	0.825
	KJ469598.1.Monkey.BR.1955	0.824	0.825
	GU001923.1.Mosquito.BR.1965	0.823	0.824
	NC_023812.1.Monkey.BR.1956	0.823	0.824
	KJ469640.1.Monkey.BR.1956	0.823	0.824
	AF159559.1.Monkey.BR.1956	0.821	0.821
	EF151503.1.Undefined.BR.1985	0.805	0.805
Lineage IV	AF159561.1.Mosquito.BR.1985	0.804	0.804
